# The potential impact of new national guidance on primary prevention of cardiovascular disease in people living with HIV

**DOI:** 10.7448/IAS.17.4.19713

**Published:** 2014-11-02

**Authors:** Nadia Ahmed, Sarah Bradley, Patrick Pearson, Simon Edwards, Laura Waters

**Affiliations:** HIV, Mortimer Market Centre, London, UK

## Abstract

**Introduction:**

Cardiovascular disease (CVD) is the leading cause of death in England and Wales. As people living with HIV (PLWH) age, proactive management of CVD risk factors is crucial. The long-awaited draft guidelines for CVD from the National Institute of Clinical Excellence (NICE) propose lipid modification (with statins) and lifestyle modification for 40–74 year olds with >10% (previously >20%) 10-year risk of CVD using QRISK2. We currently use Framingham so compared 3 CVD risk calculators in our cohort and analyzed the impact of a change in CVD threshold on the proportion of our patients who would need intervention.

**Materials and Methods:**

Framingham, QRISK2 and JBS3 cardiovascular risk calculators were compared in a group of randomly selected patients. Then, to analyze the impact of a change in primary prevention threshold on our cohort, we interrogated a prospectively collected database to identify all individuals who had a documented Framingham risk assessment and applied the current/proposed thresholds accordingly. We performed the same analysis for the three calculator subgroup (recalculating Framingham risk). Finally we surveyed HIV services in England & Wales regarding their choice of calculator.

**Results:**

We compared the 3 CVD risk calculators in 100 patients, see Table 1.

In terms of eligibility for primary prevention 20.9% (916/4383) had documented Framingham risk assessment as part of routine care. Using a 20% threshold, 8.8% (81/916) would require intervention, increasing to 35.2% (322/916) with a threshold for intervention of 10%. Restricting analysis to the 100 patients to whom we applied all three calculators resulted in the following proportion requiring intervention with a 20%/10% threshold, respectively: Framingham 28%/76%, QRISK2 20%/53%, JBS3 15%/42% (four patients were excluded due to incomplete data).

**Conclusions:**

Reducing the threshold for cardiovascular preventative measures to 10% vastly increases the number of patients requiring primary intervention, from two- to fourfold depending on risk calculator used. This may have significant implications, including cost, drug–drug interactions and patient experience, that HIV physicians and general practitioners will need to address, ideally in a coordinated and patient-focused manner.

**Table 1:** CVD risk calculator comparison

	Framingham	QRISK2	JBS3
Low/<10%	24	47	56
Medium/10–20%	48	33	25
High/>20%	28	20	15

